# The Effect of Gaze Position on Reaching Movements in an Obstacle Avoidance Task

**DOI:** 10.1371/journal.pone.0144193

**Published:** 2015-12-04

**Authors:** Alasdair Iain Ross, Thomas Schenk, Constanze Hesse

**Affiliations:** 1 School of Psychology, University of Aberdeen, Aberdeen, United Kingdom; 2 Clinical Neuropsychology, Ludwig-Maximilians-Universität, Munich, Germany; State University of New York Downstate Medical Center, UNITED STATES

## Abstract

Numerous studies have addressed the issue of where people look when they perform hand movements. Yet, very little is known about how visuomotor performance is affected by fixation location. Previous studies investigating the accuracy of actions performed in visual periphery have revealed inconsistent results. While movements performed under full visual-feedback (closed-loop) seem to remain surprisingly accurate, open-loop as well as memory-guided movements usually show a distinct bias (i.e. overestimation of target eccentricity) when executed in periphery. In this study, we aimed to investigate whether gaze position affects movements that are performed under full-vision but cannot be corrected based on a direct comparison between the hand and target position. To do so, we employed a classical visuomotor reaching task in which participants were required to move their hand through a gap between two obstacles into a target area. Participants performed the task in four gaze conditions: free-viewing (no restrictions on gaze), central fixation, or fixation on one of the two obstacles. Our findings show that obstacle avoidance behaviour is moderated by fixation position. Specifically, participants tended to select movement paths that veered away from the obstacle fixated indicating that perceptual errors persist in closed-loop vision conditions if they cannot be corrected effectively based on visual feedback. Moreover, measuring the eye-movement in a free-viewing task (Experiment 2), we confirmed that naturally participants’ prefer to move their eyes and hand to the same spatial location.

## Introduction

In order to interact with the world around us, evolution has provided humans with an extensive visual field but only with a small area of high visual acuity (the fovea). Consequently, we rely on the saccadic system that quickly directs the fovea towards the targets that guide our actions [1, 2]. Ballard and colleagues [2, 3] discovered that even in a relatively complex manipulation task, such as stacking coloured blocks, participants prefer to fixate on each object they engage with rather than relying on peripheral information. When participants were forced to fixate on a central point it took them three times longer to complete the manipulation task than when regular eye movements were allowed. Similarly, studies investigating eye movements during natural everyday tasks, such as preparing a cup of tea or making a sandwich, have shown that eye-movements typically precede motor actions suggesting that the main role of vision is to provide the motor system with the information needed to successfully complete an action [4–8]. In short, studies investigating where people preferably look when either performing simple reaching [9–13] and grasping movements [14–16], or more complex natural movement tasks [6, 17] have consistently shown that we prefer to foveate the objects we are manipulating and generally select fixation locations that are relevant for action planning and control.

Nevertheless, humans also perform many day-to-day tasks in visual periphery, such as reaching out and grasping a sandwich while simultaneously proof-reading an article. Studies investigating the accuracy with which humans are able to carry out visuomotor tasks in visual periphery have revealed some remarkable findings. When participants were able to see their hand as well as the target positioned in visual periphery, they seem to perform surprisingly accurate movements [18, 19]. This was observed for both reaching [20] and grasping movements [18] and for eccentricities up to 40 degrees of visual angle. In fact, for grasping, it was found that visuomotor performance in visual periphery is less variable and more accurate than the corresponding perceptual performance suggesting that movements made to visible objects in periphery may almost be as efficient as movements executed in central vision [18]. Interestingly, however, as soon as visual feedback of the moving hand is prevented, reaching [21–23] and grasping movements [24] were found to show systematic errors. Specifically, with no vision of their hand, participants tend to overestimate the retinal eccentricity of the targets resulting in reaching errors away from fixation. In grasping this increased uncertainty about target location was shown to become apparent with an overall increase in grip aperture size which correlates closely with the related reaching error [24]. Similar spatial errors also occur when reaching movements are performed to remembered locations meaning that neither vision of the moving hand *nor* the target is available [25, 26].

It was suggested that these gaze-dependent reaching errors reflect a misestimate of the true target position which is likely to result from an overestimation of the target distance relative to gaze [26, 27]; for a different view see [28]. Recently, however, an alternative interpretation was suggested by Dessing and colleagues [29]. In their study, they found that participants make no gaze-dependent reaching errors when pointing to remembered target locations (in darkness) when visual feedback about the position of the moving hand was provided. Based on this observation they argued that reaching errors in visual periphery are a result of misestimating the position of the hand rather than the position of the target relative to gaze as previously suggested. Thus, while until recently it appeared likely that accurate visuomotor performance in visual periphery is only possible if the movements are performed closed-loop such that potential mismatches between hand and target position can be corrected online and based on visual feedback, Dessing et al.’s study [29]implies that visual feedback about the hand alone may be sufficient to completely abolish any gaze-dependent errors.

The current study was designed to further investigate the question of whether visuomotor performance is immune to the effects of gaze position when vision of the moving limb is available. To do so, we employed a visuomotor task that has been frequently used to study the peculiarities of the human action system: the obstacle avoidance paradigm [30–35]. Using this paradigm has one considerable advantage compared to previous studies: In contrast to reaching and grasping tasks, there is no clearly defined target position in the obstacle avoidance task. That is, a possible mismatch between movement (hand) location and target location cannot as easily be corrected based on visual feedback. Importantly, correcting movements in visual periphery based on visual feedback was a viable strategy in all previous experiments that were performed with full vision of hand and target. In the obstacle avoidance task participants are asked to move their hand through a gap formed by two obstacles from a start position into a target area. We know from previous studies employing this paradigm that participants’ trajectories are highly sensitive to the position of the obstacles in the workspace (in both free-viewing and central fixation) meaning that they select movement paths that increase the safety margin between the hand and the obstacles present [30–35]. However, at the same time there is no clearly defined goal position meaning that visual feedback is considerably less effective to determine the most efficient movement path. Hence the obstacle avoidance task provides an ideal paradigm to investigate whether gaze position affects movements that are performed under full-vision but cannot be corrected based on a direct comparison between the hand and target position.

To summarise, in order to investigate whether or not gaze position affects visuomotor performance in closed-loop vision conditions, we asked participants to perform an obstacle avoidance task in four different viewing conditions: They were allowed to freely move their eyes, they had to keep central fixation (placing both obstacles into visual periphery), or they had to keep fixation on either the left or the right obstacle during movement planning and execution. We hypothesised that in a visuomotor task in which perceptual errors cannot be easily corrected based on visual feedback movement path selection will vary with gaze direction.

## Experiment 1

### Methods

#### Participants

Twenty-five graduate and undergraduate University of Aberdeen students (21 female, 4 male, age range = 18–29 years) participated in the experiment. One participant had to be excluded from the sample (final sample size of N = 24) as she did not perform the task as instructed resulting in trajectory measures that deviated by more than ±10 SEM from the average group measures. All participants were right-handed by self-report, had normal or corrected-to-normal vision and provided written consent. The experiment was approved by the local ethics committee (University of Aberdeen, School of Psychology ethical review board, approval number: PEC: 0608121725) and all participants provided written-consent before the experiment began.

#### Apparatus and stimuli

**Fig 1:**
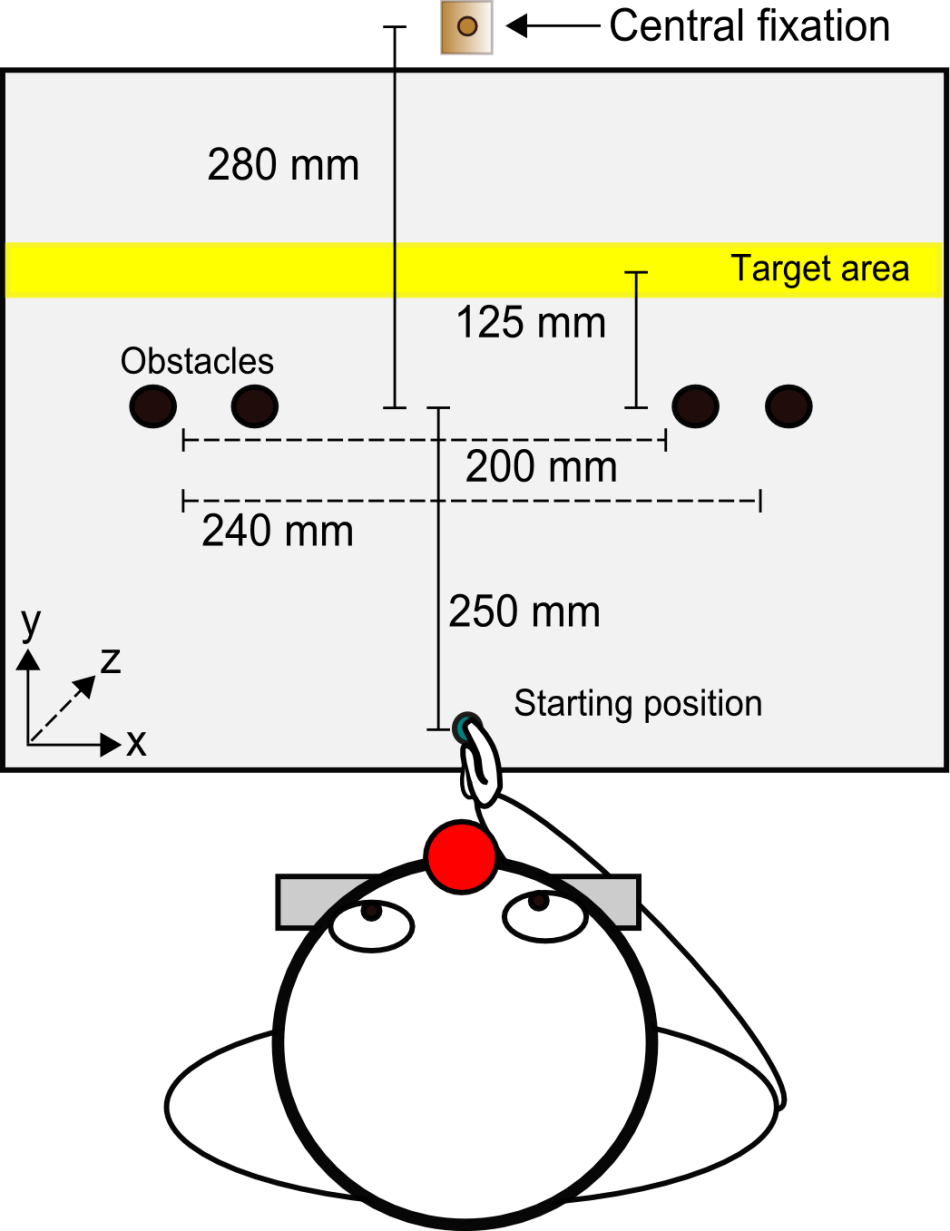
Schematic drawing of the experimental setup used in the obstacle avoidance task. Participants had to move their right-hand from the start position, between the two obstacles, into the target area.

Participants sat on a height-adjustable chair within a lit room. A wooden board (600 mm x 600 mm) was secured on top of a table and used to present the stimuli. The start position of the hand and the target zone were visibly marked out on the board (Fig 1). The start position consisted of a green felt pad (10 mm in diameter) that was aligned vertically to the midline of the participant’s body and horizontally with the middle of the board. Participants were asked to keep their hand upright with their index finger placed on top of the start position. A chinrest was used to maintain a constant head position throughout the experiment.

A strip of yellow card (600 mm x 50 mm) marked the target zone that was at a straight-line distance of 375 mm from the hand’s starting position. The obstacles were placed along a virtual horizontal line 250 mm in front of the start position (see Fig 1). The obstacles were grey cylinders (thin wooden poles wrapped in piping foam) with a height of 240 mm and a diameter of 40 mm. They could be placed at an outer or inner position forming either a gap (defined as the distance between the inner edges of the obstacles) of either 240 mm (with both obstacles at the outer-most position) or 200 mm (with one obstacle being moved 40 mm inward from the left or right side while the other remained at the outer-most position). Strips of white laminated card covered any holes where obstacle positions were marked (for similar procedure see [31]). A fixation point was placed on each obstacle with a strip of blue tape (10 mm in width, positioned at a height of 160 mm on the obstacle) with a vertical black line representing the exact fixation location. A central fixation point was placed 280 mm behind the obstacle locations, aligned centrally with the start position, and elevated 160 mm above the obstacle board. The fixation point was located on a wooden rod with a wooden base (40 mm x 40 mm); again blue tape and a vertical black line indicated the exact fixation location.

Hand position was recorded with an infrared-based Optotrak 3020 system (Northern Digital Incorporation, Waterloo, Ontario, Canada) at a sampling frequency of 200 Hz. An infrared light emitting diode (IRED) was attached to the right hand on the tip of the index finger. Prior to running the experiment, the plane of the obstacle board was calibrated to the Cartesian (x, y, z) coordinate system with the start position being set to the origin of the coordinate system. The experiment was programmed using MATLAB and the custom-built Optotrak Toolbox [36].

Fixation was monitored with a BlueGain electro-oculogram (EOG) amplifier (Cambridge Research Systems. Kent, England). Two electrodes were placed around the left eye with one being attached above right edge of the left eyebrow (top right of the superior orbital margin) and the other one below the eye toward the left temple (near the outer canthus). An additional earth electrode was attached to the left earlobe. Pilot-tests had indicated that with this setup we could reliably detect vertical and horizontal eye-movements if they exceeded about 2 degrees of visual angle resulting in voltage changes between 10–20 microVolts (depending on skin condition, tiredness etc. of participants).

EOG and Optotrak were synchronised using an infrared signal transmitted to the EOG at the beginning of each trial. The EOG data was monitored in real-time by the experimenter. If a participant failed to maintain fixation the trial was discarded and repeated at a random position within the experimental block (on average participants lost fixation in less than 7% of all trials).

#### Procedure

All participants took part in four experimental conditions systematically varying their gaze position: In the free-viewing condition (FV) participants obtained no instructions on where to look whilst performing the task. In the central fixation condition (Central-Fix), participants were asked to keep fixation at the central fixation point placed behind the obstacles throughout the trial. Finally, in both the left obstacle fixation (Left-Fix) and the right obstacle fixation (Right-Fix) conditions, participants were instructed to fixate at the fixation point placed on the left or right obstacle respectively, throughout the trial. Viewing conditions were blocked and counterbalanced across participants. The obstacle positions (both out, left in, right in) were allocated randomly within each block. Each obstacle position was presented 8 times resulting in 24 trials per block and 96 trials in total. All participants performed three practice trials prior to the start of each block.

At the start of each trial participants positioned their right hand at the start position and were asked to close their eyes. The experimenter then arranged the obstacles and manually started the trial with a key press. Subsequently, an auditory signal (500 Hz, 100 ms) indicated for participants to open their eyes and to fixate at the current fixation position (or to freely move their eyes in the FV condition). Following a 1.5 s preview period another beep (1000 Hz, 100 ms) signalled participants’ to start their reaching movement and to quickly move their hand between the obstacles into the target zone, touch the yellow card board, and move back to the start position. In all fixation conditions, participants were instructed to maintain fixation at the fixation position throughout their movement. After 3 s the Optotrak stopped sampling and participants were instructed to close their eyes again so that the experimenter could prepare the next trial.

#### Data analysis

The IRED on the index finger was used to determine the hand position throughout each trial. Movement onset was defined when the index finger had surpassed a velocity threshold of 0.025 m/s. Reaction time (RT) was defined as the time between auditory go-signal and movement onset. A combined position and velocity criterion was used to define the end of movement: Firstly, we determined when the index finger marker was in close spatial vicinity of the target zone (all frames in which the marker was within 5 mm from the furthest distance measured in y-direction) and secondly we searched for the data point with the lowest velocity within these frames. Movement time (MT) was defined as the time between movement onset and end of movement. Across all participants trials with RTs below 100 ms (movement onset before the auditory go-signal) and trials with missing data were excluded from analysis (a total of 8 trials).

Furthermore, we calculated time-normalised movement trajectories by dividing the data between movement initiation and end of movement into 100 equal time intervals using linear interpolation. Additionally and in accordance with previous studies, we determined the lateral position (x-direction) of the maker at the moment the index finger passed between the obstacles in y-direction (see also [31–35]).

### Results

**Fig 2:**
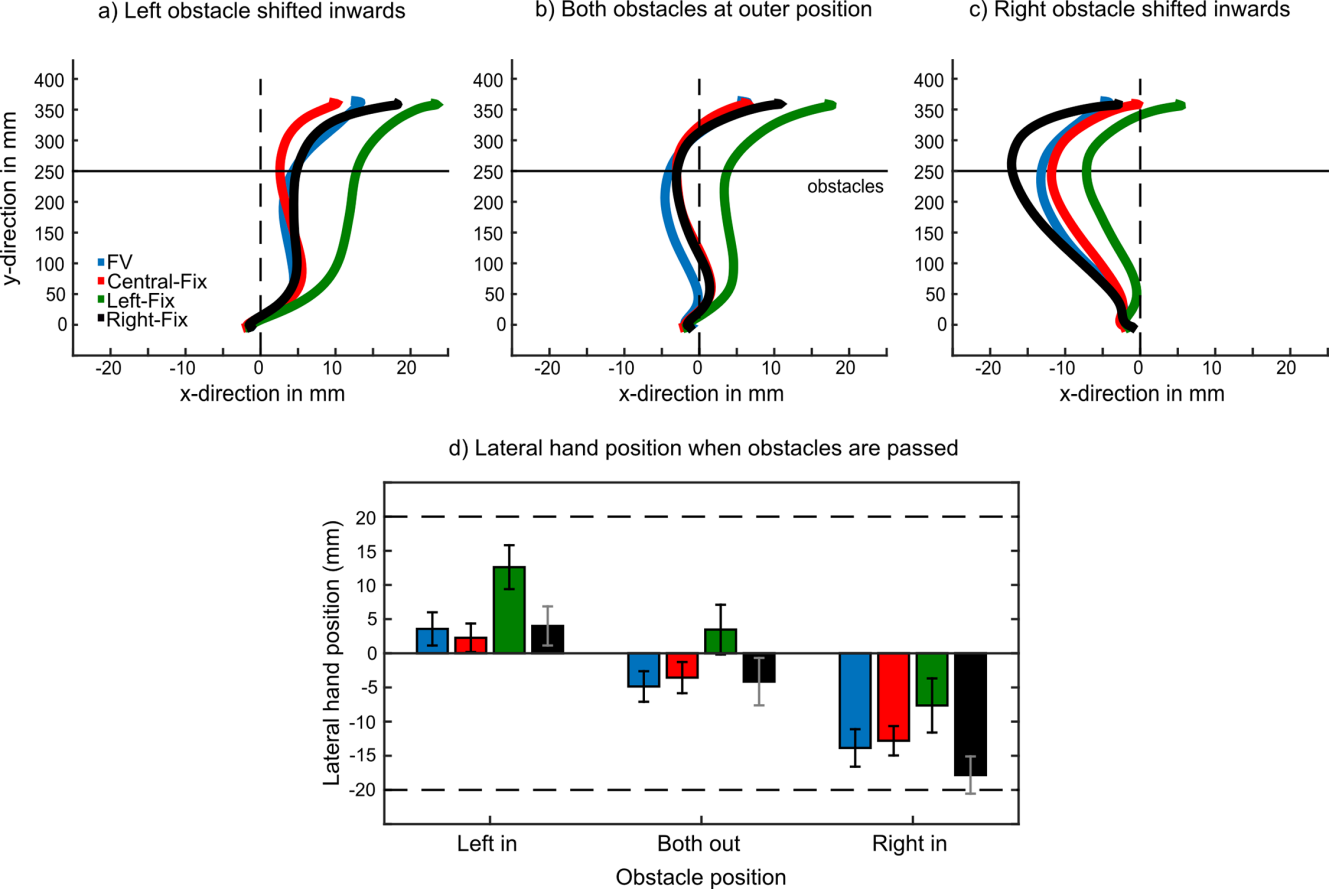
Experiment 1. a-c): Mean time-normalised trajectories for all 24 participants for each gaze condition when a) the left obstacle was shifted inwards, b) both obstacles were at the outer position, and c) the right obstacle was shifted inwards. d) The mean lateral hand position at the moment the obstacles were passed as a function of obstacle configuration and gaze condition. A position to the left of the midline results in a negative value and a position to the right a positive value. Error bars depict ± 1 SEM (between subjects).

In order to test if gaze position affected movement path selection, we calculated the average time-normalised trajectories for each gaze condition and obstacle configuration (Fig 2A–2C). Visual inspection of these figures suggests that participants selected a movement path further to the right of the midpoint when the left obstacle was fixated and slightly further to the left when the right obstacle was fixated. To determine statistically if there was an effect of fixation position on the hand position, we determined the lateral position of the hand at the moment the obstacles were passed (Fig 2D).

The 4 (gaze condition: FV, Central-Fix, Left-Fix, Right-Fix) x 3 (obstacle position: both out, left in, right in) repeated-measures ANOVA on this data confirmed, as expected, a significant main effect of obstacle position, F(2,46) = 205.13, ε = .605, p < .001, suggesting that participants selected different trajectories depending on position of the obstacles. When both obstacles are presented at the outermost position the midpoint is located at 0 mm, the midpoint shifts to ±20 mm when one of the obstacles is moved inwards (negative values when the right obstacle is moved and positive values when the left obstacle is moved). The mean lateral position of the hand across all gaze conditions, when both obstacles were presented at the outermost position, was -2.3 mm ± 1.3 mm. When the left obstacle was moved inwards the average lateral position of the hand was at +5.6 mm ± 1.3 mm, and when the right obstacle was shifted inward, it was at -13.0 mm ± 1.3 mm. Post-hoc tests confirmed that the differences between all obstacle positions were highly significant (all p < .001). The finding that shifting the left obstacle from an outward to an inward position results in smaller trajectory adjustments (7.9 mm ± 0.4 mm) than a shift of the right obstacle (18.6 mm ± 1.2 mm) is consistent with previous studies on obstacle avoidance [30, 35, 37–39] and is likely to be related to the fact that the right obstacle is more obstructive for right-handed movements than the left obstacle.

More importantly, however, we also found a main effect of gaze condition, F(3,69) = 6.00, ε = .598, p = .007. Post-hoc tests revealed that participants passed the obstacles further to the right of the midline when the left obstacle was fixated (Left-Fix) as compared to all other conditions (all p < .02). None of the other comparisons were significant.

Finally, the ANOVA also revealed a significant interaction effect between gaze condition and obstacle position, F(6,138) = 3.45, ε = .590, p = .015. This interaction effect indicates that participants’ lateral hand position in the three obstacle configurations varied depending on the gaze condition.

**Fig 3:**
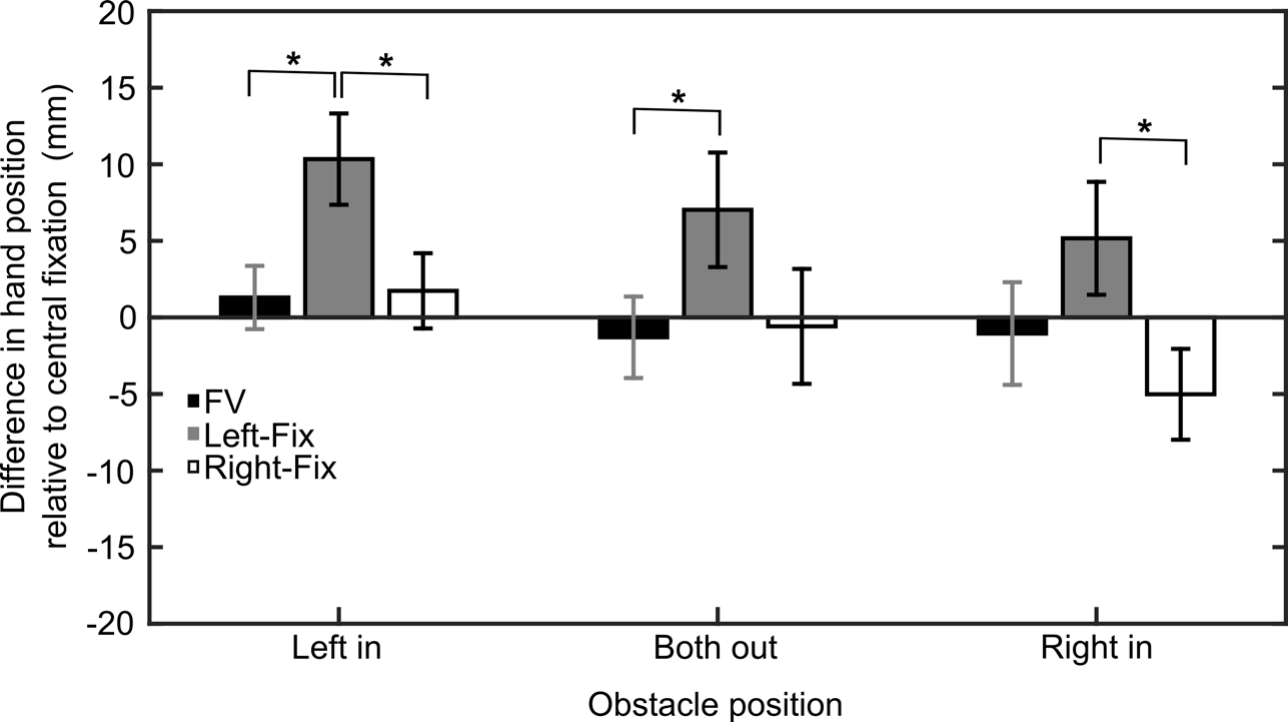
Experiment 1. Mean difference in lateral hand position between Central-Fix and the other gaze conditions at the moment the obstacles were passed as a function of obstacle configuration. Positive values indicate a hand position to the right of the one measured in the central fixation condition and negative values a hand position to the left of the one measured during central fixation. Error bars depict ± 1 SEM (between subjects).

In order to further explore this interaction effect, and thus the question of how gaze position modulates the influence of obstacle position on reaching, we calculated the lateral hand position in all gaze conditions relative to the central fixation condition (in which both obstacles were presented in visual periphery). To achieve this, we computed the differences between the average lateral hand positions (at the moment the obstacles were passed) in the gaze condition in question (e.g., Right-Fix, Left-Fix, FV) and the respective value obtained in the central-fixation condition for each obstacle position and participant. This procedure has the advantage that baseline changes in hand-position (as measured in central fixation) are removed and we can thus explore how shifts in gaze-position, relative to this baseline, modulate the effect of obstacle positions. The difference values obtained for each gaze condition are depicted in Fig 3.

A 3 (gaze condition: FV, Left-Fix, Right-Fix) x 3 (obstacle position: both out, left in, right in) repeated-measures ANOVA on these difference values revealed a significant main effects of gaze condition, F(2,46) = 6.74, ε = .665, p = .009, and obstacle position, F(2,46) = 9.28, p = .001, but no interaction effect (p = .13). These findings indicate that both gaze condition and obstacle position have distinct effects on hand position relative to central fixation. Please note that the main effect of obstacle position is owed to the fact that in the left-in condition the hand position is for all gaze conditions further to the right (positive values) than in the central-fixation task while in the right-in condition the effects for the different gaze conditions go in opposite directions (positive and negative values) compared to central fixation (see Fig 3). Therefore, since the effects of left and right fixation cancel each other out in the right-in condition, the main effect of obstacle position (misleadingly) suggests that the hand position is more strongly affected by eccentric viewing in the left-in condition than in the right-in condition. Considering that the effects of obstacle position (and their direction) are dependent on the gaze condition we calculated three one-way ANOVAs, one for each obstacle position, with gaze condition as the independent variable. There was a significant effect of gaze condition when the left obstacle was moved inward, F(2,46) = 8.13, ε = .787, p = .002. This effect was mainly caused by the fact that, relative to central fixation, the hand position was further to the right when the left obstacle was fixated than when free-viewing was allowed (p = .001) or the right obstacle was fixated (p = .01). Similarly, when both obstacles were at the outer position, the effect of gaze condition, F(2,46) = 4.95, ε = .693, p = .023, indicated that the hand position was further to the right when the left obstacle was fixated as compared to the free-viewing condition (p = .004). Finally, when the right obstacle was moved inward, there was again an effect of viewing condition, F(2,46) = 5.60, ε = .804, p = .011. Post-hoc tests confirmed that there was a significant difference between left and right obstacle fixation with the hand position being further to the left when the right obstacle was fixated (p = .012).

Finally, we also investigated if participants’ RTs and MTs varied dependent on gaze and obstacle condition. A 4 (gaze condition: FV, Central-Fix, Left-Fix, Right-Fix) x 3 (obstacle position: both out, left in, right in) repeated-measures ANOVA revealed no significant main effects or interactions neither on RT nor on MT data (all p > .18). On average, it took participants 254 ms ± 8 ms to initiate their movements and 720 ms ± 27 ms to execute their movements. Hence, neither movement initiation time nor movement execution time was increased due to peripheral viewing conditions.

## Experiment 2

In Experiment 1, we found that when participants fixated the left obstacle they tended to move their hand further away from it (i.e. to the right) as compared to the free viewing or central fixation conditions. Even though, participants showed a small tendency to move their hand further to the left when fixating the right obstacle (see Fig 2) this finding did not reach significance. As we observed very little difference between free-viewing and the right obstacle-fixation gaze condition, we wondered if participants naturally look at the right obstacle when performing the task without restrictions. This could possibly also explain why we observed more pronounced effects on hand position when the right obstacle was shifted from an outer to an inner position than when the left obstacle was moved inward. Making selective eye-movements to the right obstacle could be a sensible strategy as it is the obstacle that is placed closer to the moving arm and thus more obstructive to the movement. In order to test where participants preferably look when performing the obstacle avoidance task we conducted an additional experiment in which we tracked participants’ eye-movement in a free-viewing condition.

## Methods

### Participants

Seventeen students (13 female, 4 male, mean age = 23 years, range = 18–41 years) participated in the experiment. Two participants had to be excluded due to difficulties in calibrating the eye tracker (final sample size of N = 15). All participants were right-handed by self-report, had normal or corrected-to-normal vision and provided written consent. The experiment was approved by the local ethics committee (University of Aberdeen, School of Psychology ethical review board, update on approval number: PEC: 0608121725) and all participants provided written-consent before the experiment began.

#### Apparatus and procedure

The experimental setup was similar to Experiment 1 (see Fig 1). A chinrest with a fixed height of 380 mm was used such that the centre of the eyes was elevated approximately 450 mm above the obstacle board. As we were interested in examining participants’ free-viewing behaviour we removed all the fixation points from the obstacles as well as the central fixation rod.

Hand trajectories were recorded using an infrared-based Optotrak 3020 system with an IRED attached to the index finger of the right hand. Binocular gaze was monitored in real-time using SMI eye-tracking glasses (SensoMotoric Instruments, Teltow, Germany). Individual cameras recorded participants’ field-of-view and eye position at a rate of 60 Hz (pupil tracking accuracy about 0.5° of visual angle). In total there were 30 trials, with 10 trials for each of the three possible obstacle positions. Prior to the start of the experiment, participants performed three practice trials.

At the start of each trial, they positioned their right hand at the start position and were asked to close their eyes. The experimenter then arranged the obstacles and manually started the trial with a key press. In response to an auditory signal (500 Hz, 100 ms) participants were instructed to open their eyes. After a 1.5 s preview period, another beep (1000 Hz, 100 ms) indicated to the participants to start their movement. Similarly as in Experiment 1, participants were instructed to quickly move their hand between the obstacles into the target zone.

#### Data analysis

Movement data was analysed in the same way as in Experiment 1. For the eye movement data, video images were viewed in frames (for each trial) using MATLAB. Frames of interest (the point at which the index finger reached the obstacles) were manually coded (i.e. gaze position, obstacle position and finger position were determined in 2D pixel coordinates within MATLAB). As the true distance between the obstacles (gap size) was known, gaze position could be calculated as a percentage of this distance and transferred from pixel into mm allowing a direct comparison between gaze position and hand position. Across all participants trials with missing data (hand trajectories/eye movements) or trials with RTs of less than 100 ms were excluded from analysis (a total of 18 trials across the whole experiment). A one-factorial (obstacle position: both out, left in, right in) repeated-measures ANOVA was used for statistical analysis.

To remain consistent with Experiment 1, we determined the lateral position of the hand as well as the gaze position at the moment the obstacles were reached. However, as the gaze data suggested that participants primarily looked at the target region throughout the reach we additionally analysed the lateral hand position at the end of movement and compared it with the gaze position determined at the moment the obstacles were passed.

### Results

**Fig 4:**
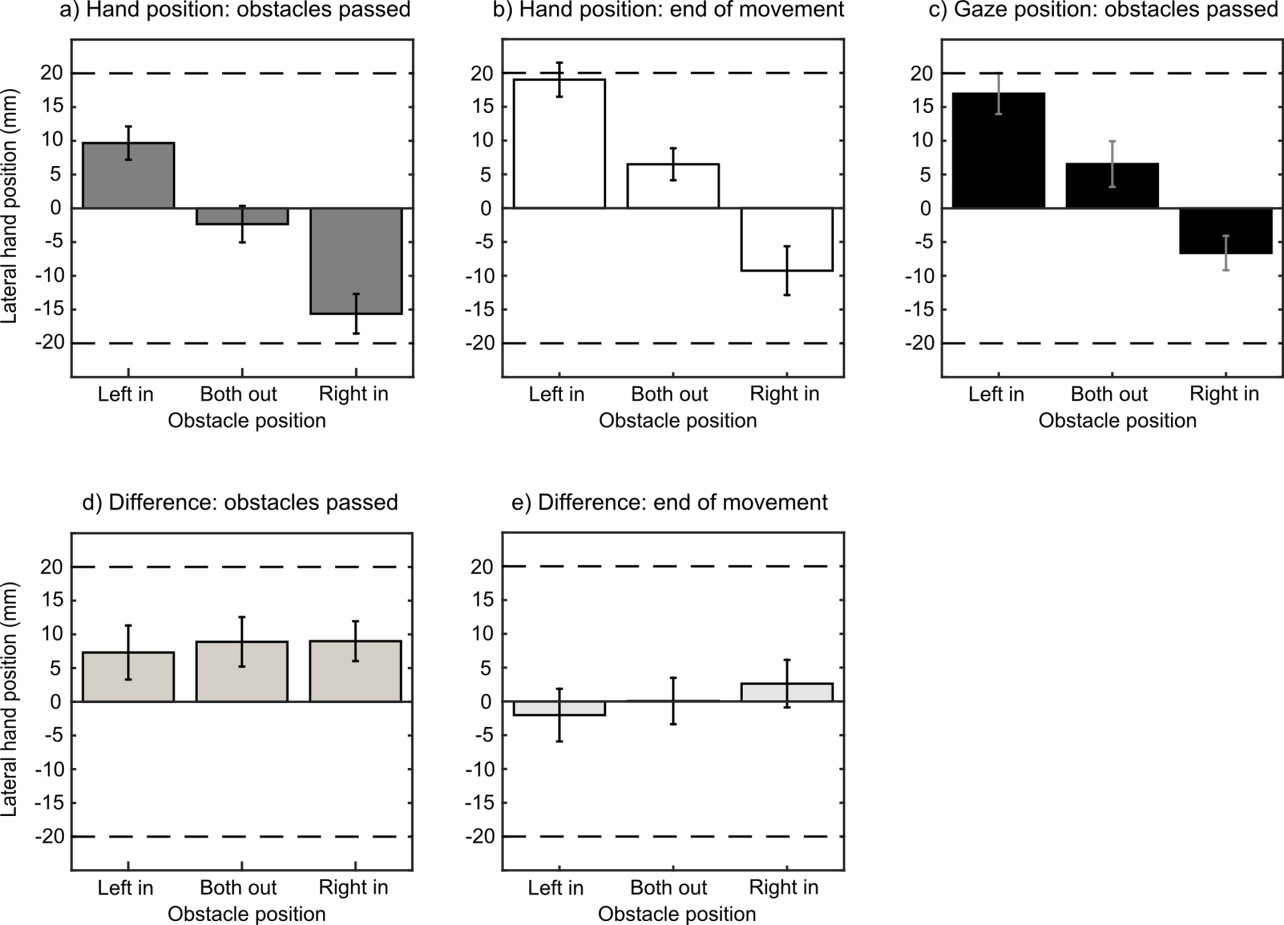
Experiment 2. a) Mean lateral hand position at the moment the obstacles were passed. b) Mean lateral hand position at the end of movement. c) Mean lateral gaze position at the moment the obstacles were passed. A position to the left of the midline results in a negative value and a position to the right a positive value. d) Mean difference between gaze position and hand position at the moment the obstacles were passed. e) Mean difference between gaze position and hand position at end of movement. Positive values indicate that the fixation position was to the right of the index finger. Error bars depict ± 1 SEM (between subjects).

Similar to Experiment 1, we expected consistent changes in the lateral hand position depending on the positioning of the obstacles. In accordance with this prediction, a one-way (obstacle position: both out, left in, right in) repeated-measures ANOVA revealed a highly significant main effect of obstacle position on the lateral hand position, F(2,28) = 59.92, ε = .641, p < .001. The mean lateral position of the hand when both obstacles were presented at the outermost position was -2.3 mm ± 2.7 mm. When the left obstacle was shifted inwards the average position of the hand was +9.7 mm ± 2.5 mm to the right of the midline, and when the right obstacle was shifted, it was -15.6 mm ± 2.9 mm to the left of the midline (Fig 4A). Paired samples t-tests indicated that all differences between the obstacle positions were highly significant (all p < .001).

On average participants fixated the target area in 94% of trials at the moment the obstacles were passed. In the remaining 6% of the trials fixations fell on the hand passing the obstacles. A one-way repeated-measures ANOVA (obstacle position: both out, left in, right in) on the lateral gaze position data revealed that participants lateral gaze position varied depending on the obstacle position, F(2,28) = 32.09, p < .001. When both obstacles were placed in the outermost position the mean lateral gaze position was biased slightly towards the right of midline (+6.6 mm ± 3.4 mm). When the left obstacle was shifted inwards the average gaze position was at +17.0 mm ± 3.0 mm, and when the right obstacle was shifted inward the gaze position was -6.6 mm ± 2.5 mm to the left of the midline (Fig 4C). Paired samples t-tests confirmed that all differences between obstacle positions were significant (all p < .012).

The data suggests that the lateral gaze position (when obstacles were passed) was right to the position of the index finger at the moment the obstacles were passed. As the trajectories determined in Experiment 1 suggest that participants tend to move their hand further to the right between passing the obstacles and reaching the target zone (see Fig 2A–2C), the corresponding lateral gaze position (that was mostly directed at the target area) may reflect the predicted/targeted landing position of the index finger (see Fig 4B). To test this, we calculated the difference between lateral gaze position and lateral hand position at the moment the obstacles were passed (Fig 4D) as well as the difference between lateral gaze position the moment obstacles were passed and the lateral hand position at the end of the movement (Fig 4E) for all obstacle configurations. As there was no effect of obstacle position on the differences between gaze and hand position (p = .86 when obstacles were passed; and p = .47 at end of movement), data was averaged across all obstacle configurations. One-sample t-tests confirmed that the gaze position was to the right of hand position when the obstacles were passed (8.4 mm ± 3.0 mm; p = .014). In contrast, at the end of the movement, the difference between lateral hand and gaze position was not different from zero (0.2 mm ± 2.9 mm; p = .94) indicating that hand and gaze position were identical.

In contrast to our assumption, we did not find that participants fixated more frequently on the right obstacle than the left one. In fact, at the moment the hand reached the obstacles, none of our participants were ever looking at them. However, focusing on the point in time, when the obstacles are passed, leaves open the possibility that participants fixated the obstacles before initiating movement, or prior to reaching them. To test this possibility, we computed for every trial whether and how often participants looked at the obstacles during the interval from opening the eyes until the end of movement (the target strip was touched). Interestingly, we found that only 2 out of the 15 participants ever looked at the obstacles during this period. Both of them looked at the obstacles during the 1.5 s preview period after the eyes were opened but before the movement was initiated (one person in 8 out of 30 trials and the other person in 12 out of 30 trials). These fixations were spread evenly between the left and right obstacles and did not appear to be influenced by obstacle position.

## General Discussion

The aim of the current study was to investigate whether movement path selection varies with gaze position when full vision of hand and target is available. To this end, we employed an obstacle avoidance paradigm in which participants had to move their hand through a gap formed by two obstacles, which could be placed at different locations. In the first experiment we used four different viewing conditions: free-viewing, central fixation (placing both obstacles in visual periphery), or fixation on either the left or right obstacle (placing one of the obstacles in visual periphery). As expected, participants reliably adjusted their trajectories in response to the obstacle positions in all gaze conditions. More importantly, however, we found evidence that gaze position affects movement path selection when avoiding obstacles. Specifically, participants moved their hand further away from the left obstacle if it was fixated while fixations on the right obstacle had no distinct effects relative to free-viewing and central fixation conditions.

Previous reports investigating the effect of gaze location on hand movements seem to indicate that whether or not humans are able to perform accurate actions in visual periphery depends on the availability of visual feedback. Studies that investigated open-loop (and memory guided) reaching and grasping movements, performed to targets presented in visual periphery, found that participants tend to systematically overestimate target eccentricity [21–23, 25, 26]. However, when full vision of hand and target is available (closed-loop movements) visuomotor performance remains very accurate even at large eccentricities [18, 19, 40]. As it is well-known that visual feedback, aids the correction of movements [41–43], we wished to investigate if movements, performed visually-closed loop, are affected by gaze position if errors cannot clearly be identified based on a perceptual mismatch between hand and target position.

By employing an obstacle avoidance task, we ensured that we used a visuomotor paradigm that has no clearly defined target position rendering the available visual feedback less useful. While our findings show that gaze-position substantially affects visuomotor performance, the source of those gaze-dependent errors is less clear. However, we do think that our findings can help to mediate between two different accounts. Traditionally, it was argued that the source of the error is an overestimation of the distance to peripheral targets [26, 27]. In the case of reaching this will lead to overshooting reaching movements. In the case of our obstacle avoidance paradigm, it would mean that the distance to the obstacle viewed in visual periphery would be overestimated leading to a shift away from the fixated obstacle towards the position of the peripheral obstacle. This is exactly what we observed when the left obstacle was fixated but only found to a very marginal extent when the right obstacle was fixated. The asymmetry between left and right obstacle fixation may be due to the fact that all of our participants reached with their right arm. Therefore the right obstacle was more obstructive for the movement, as it was placed on the same side as the forward-reaching arm (for similar findings see [30, 35, 37, 38, 44]). The closer the obstruction is to the movement path, the more relevant it becomes for movement planning. Accordingly, errors in the estimated distance of the right eccentric object (left obstacle fixation) will have a more profound effect on hand position than a misestimate of the position of the left obstacle (right obstacle fixation). Thus, all-in-all our findings fit nicely with the idea that the position of the peripheral obstacle is overestimated.

However, there is also a different suggestion. Dessing et al. [29]argued that it is not the targets (or in our case the obstacles) whose positions are incorrectly estimated but the hand-position. In principle such an assumption can just as easily explain our findings. If our participants underestimate the distance between fixation-position and hand-position the same effects are expected. For example, in the case where participants fixate the left obstacle they might assume that the hand is closer to the left obstacle than it actually is and consequently shift the hand away from the left obstacle and towards the right (peripheral) obstacle. Yet, the claim made by Dessing et al. [29] is more specific. They argue that the error arises during the transformation of the proprioceptive hand-signal into gaze-centred coordinates. Based on this assumption they predict that if the transformation is made superfluous by providing a visual hand-position signal, the gaze-dependent error disappears. They confirmed this prediction in a study on memory-guided reaching. Applied to our case, in which vision of the hand was available, it would consequently be predicted that as no proprioception-to-vision transformation is necessary, no concomitant gaze-dependent error should occur. Clearly this was not the case in our experiment. It remains unclear whether it is the positional coding of the obstacle or hand that is affected by gaze-position. However, our findings clearly suggest that at least for immediate reaching the proprioception-to-vision transformation is not the sole or most dominant source of gaze-dependent reaching errors.

Finally, our findings also make a noteworthy contribution to the discussion regarding the nature of the obstacle avoidance task. As, during obstacle avoidance, participants tend to move their hands through the middle of the gap, it was argued that this task may represent a visuomotor equivalent of a perceptual line-bisection task in which participants are asked to indicate the mid-point of lines varying in length [32–34]. However, as we demonstrated recently, there are critical differences between the two tasks as obstacle avoidance, but not line-bisectioning, was found to be critically influenced by the initial starting position of the hand [35]. Based on these findings, we suggested that obstacle avoidance and line-bisectioning are less alike than previously assumed. The current study further supports this notion as studies that systematically varied the fixation position of participants in the perceptual line-bisection task actually found exactly the opposite effect of gaze positon on line-bisection accuracy [45, 46]. That is, when participants had to keep eccentric fixation (such that the line was only visible in one hemifield) they showed a consistent bisection error towards fixation (i.e. shifting the perceived midpoint closer to the fixation point). It was suggested that this bias might be due to an overestimation of the portion of the stimulus located closer to the fovea (central magnification, see [45]). If, however, similar mechanisms underlie obstacle avoidance and line-bisectioning a comparable bias toward the central visual field should also become apparent in the obstacle avoidance task. The fact that we found a bias in the opposite direction, i.e. away from fixation, further supports the suggestion that participants apply different strategies during line-bisectioning and obstacle avoidance (for further discussion of this issue see [35]).

However, before we conclude, we have to consider a possible alternative explanation for our findings. In principle, it could be argued that the effect of gaze-position may be due to an increased saliency of shifts of the fixated obstacle. As fixation position was blocked, it is possible that participants tried to keep fixation at the obstacle between trials (whilst having their eyes closed). In this case, if the fixated obstacle is moved, participants will need to adjust their fixation after opening their eyes making the obstacle shift more salient and simultaneously providing oculomotor information about its size. If, for example, a participant fixates at the left obstacle and the left obstacle is shifted, then this shift becomes salient and the adjustment in hand position should be more pronounced (relative to the adjustments made in central fixation). More specifically, if we analyse the left-obstacle fixation condition and look at the left-in condition, then either there was no shift of this obstacle or, if there was, it had been moved inwards. In contrast, if we look at the baseline condition (both out), then either the left obstacle remained at its position or there had been an outward shift. Therefore, if the left obstacle was fixated, an inward shift should be more salient in the left-in condition (resulting in a hand position further to the right than in all other gaze conditions) and an outward shift more salient for the both-out condition (resulting in a hand position further to left than in all other gaze conditions). As we observed a) a consistent rightward bias for all obstacle configurations when the left obstacle was fixated and b) no interaction effect between obstacle position and gaze condition (when analysing the data relative to central fixation), we think that an increased saliency of the shift-direction for the fixated obstacle is unlikely to account for the gaze-on-hand-position effects. Yet, it was shown in a recent study by Menger et al. [47] that solely drawing participants attention to one of the obstacles (using a flashed LED) can result in adjustments of the hand away from this obstacle. These attentional effects were limited to the right obstacle, again confirming that right-handed movements are more sensitive to the position of the right obstacle. Given that there is a close connection between fixation position and spatial attention [48], it stands to reason that in our paradigm spatial attention may have been biased toward the obstacle fixated. Ultimately, the question of whether our findings have a merely perceptual origin or are mediated by attentional factors needs to be clarified in future studies.

Finally, in order to test more directly, if and how eye and hand movements are coupled in the obstacle avoidance task, we ran a second experiment in which we monitored participants’ eye-movements in the free-viewing condition. Consistent with the idea that eye-movements are mainly directed toward the target location of the movement, we found that (lateral) gaze position varied in line with the (lateral) hand position for the different obstacle configurations. At the moment the obstacles were passed the gaze was already directed at the target area at a lateral position close to where the hand was going to land. That is, in most trials, the lateral gaze position at the moment the obstacles were passed coincided with the final lateral landing position of the hand. Surprisingly, only two out of the fifteen participants ever looked at the obstacles before movement initiation in some of the trials. These findings suggest that even in the absence of an explicit movement target, participants select a given target area and presumably use this location to guide their reaches. Generally, our observation that participants primarily fixate the target of their movement but rarely look at obstacles, their body parts or objects irrelevant for the task is in line with previous studies investigating obstacle avoidance tasks in grasping [14] or walking [49, 50].

## Conclusion

In this study we investigated whether fixation position affects the selection of the movement path in an obstacle avoidance task. Our findings indicate that hand position varies depending on where participants are instructed to look when moving their hands. Generally, participants show a tendency to select movement paths that veer away from the obstacle they are looking at. In accordance with previous research, we observed that when gaze is unrestricted, participants primarily make fixations in the target area close to the position they are aiming at with their hand. While numerous studies have already shown that people naturally tend to look at the target location of their movement, our study provides evidence that fixation location in turn affects movement path selection. We suggest that these effects are primarily perceptual in nature and can probably be attributed to a misjudgement of the position of the obstacle placed in visual periphery. However, we cannot completely exclude the possibility that the effects are moderated by attentional mechanisms that are closely linked to fixation location. Nevertheless, our findings are valuable as they indicate that differences in visuomotor performance may in certain cases be attributed to the underlying fixation pattern.
